# Notes and Comments

**Published:** 1923-03

**Authors:** 


### NOTES AND COMMENTS.

### THE NEW HOUSING PROGRAMME.

THE country is now in possession of an outline of the Government's housing policy, though the details are as yet something less than clear. In so far as its proposals for the early de-control of rents are concerned, the Report of the Onslow Committee is rejected, or its dates postponed, less, it would appear, on its merits than in deference to political and Parliamentary exigencies. Its proposals for gradual de-control between now and the middle of 1925 were entirely reasonable in themselves, since it is certain that until de-control comes and free trade in house-building is restored, we shall continue to move in a vicious circle. Private capital will not flow freely into bricks and mortar until it is once more untrammelled. The Government propose to encourage building by a modified form of subsidy, representing half the loss, payable to the local authorities, who may either build themselves or pay the subsidies to others. These grants in aid will be payable only in respect of the smallest type of house—the type most urgently needed. Apparently there is to be no de-control until the summer of next year, while the operation is to be completed at a later date, by which time it is hoped that a sufficient number of dwellings will have been provided. The hope is vain. There will, no doubt, be a good deal of building, but it is inevitable that it should be woefully insufficient. Then there will be a demand for the further maintenance of rent restriction, and so the vicious circle will continue.

### POLITICS AND HOUSE-BUILDING.

IT is a most unhappy circumstance that politics should have been allowed to enter into that portion of the economic domain which is represented by house-building. A cry has been raised that the withdrawal of restrictions upon the rents of the larger type of middle-class houses would be followed by ruthless increases of rent, and the Labour Party has flown to the succour of that *bourgeoisie* with which it commonly exhibits little sympathy. This is a humorous incident, since it is the admitted policy of labour not only to eliminate the middle classes but to make impossible the private ownership of property. That is why the minority report of the Departmental Committee advocated not only the continuance of control until 1930, but the reduction of the rents authorised by the last Rent Restriction Act. The characteristic failing of modern Governments everywhere is timidity, the orthodox name for which is, we believe, electoral prudence. But if the housing difficulty is ever to be solved, some Cabinet will have to be courageous enough to get off the spiky fence on to hard ground.

### RÖNTGEN AND NORDAU.

DURING the past month two outstanding personalities of widely different types have passed away—William Conrad von Röntgen and Max Nordau. The one was a constructive genius in the sense that he added powerfully to our means of dealing with disease; the other was, if anything, a destructive force. Alike in surgery and in medicine, Röntgen's discovery of the X-rays and their remarkable properties has conferred the highest benefits upon humanity. He has left a bright and permanent mark upon the modern history of curative science, and has proved once more that the researches of the physicist are full of those possibilities for romance which obtain their highest expression in the removal or prevention of disease. Such a man as Röntgen is essentially an optimist; Max Nordau was essentially a pessimist. As an analyst of social maladies he was the sworn foe of most things that are, of most things to which experience has led mankind to attach importance. To him religion, marriage, monarchy were merely the "Conventional Lies of Our Civilisation." Such work may have its uses by helping to startle the world out of its complacency; but in the nature of things it is fugitive. Nordau, who, like Röntgen, was a physician, was neither prophet nor seer, yet he was combative and consequently stimulating and had an admirable method of setting the thoughtless to the woeful task of thinking, even if they thought wrong.

### SIR WILLIAM TRELOAR.

FEW Lord Mayors of London have created so splendid a memorial of their twelve months' reign as Sir William Treloar, who, in celebration of his philanthropic work, has just been given a luncheon at the Mansion House. The Cripples' Hospital at Alton Park in Hampshire, which he established in 1907, was sorely needed and has filled a bad gap in our hospital system. At that time the condition of children crippled by tuberculosis was pitiable and called loudly for practical help, and the £60,000 which Sir William Treloar collected by a Mansion House Fund was used for the equipment of the Alton Hospital which had been left derelict after the departure of the last of the men wounded in the South African War. There is something peculiarly pathetic about the fate of a child unfitted by tuberculosis for struggling with a world in which only the healthy and virile enjoy a real chance of success. The Alton Hospital has provided that opportunity for great numbers of children, and to have done that is to entitle the doer to the grateful thanks of a community which has been enriched by his work.

### CO-ORDINATION OF RELIEF.

EVERYONE who has practical knowledge of the extent to which our State Benefit Schemes are in danger of overlapping, or do actually overlap, will learn with satisfaction that the Prime Minister has appointed a Departmental Committee to investigate the whole subject. The Ministry of Health is more or less closely responsible for the whole of these benefits, whether by the Ministries of Pensions and Labour, or by the Poor Law authorities. During the current financial year something like three millions a week are being paid in pensions, medical and sickness benefit and unemployment pay, and it is almost inevitable that with so many authorities administering these various reliefs there should be overlapping, sometimes to a serious extent. A single controlling authority in each district would seem to be the obvious way out of this particular difficulty, though the farther one, of how long we shall be able to continue relieving a large proportion of the population on the present scale, will not so easily be solved. Happily, the reduction in the number of the wholly, or partially, unemployed which continues to take a substantial number of men and women off the "live registers" every week, is progressively lessening the gravity of the problem.

### DISPOSAL OF REFUSE.

WITH the exception of certain of the great towns, our methods of disposing of household refuse are still elementary. It is difficult to teach householders that they ought to burn much of the rubbish which they at present throw into the dust-bin, but until they have learned this elementary lesson in sanitary science the volume of matter to be dealt with will always create difficulty. Of late many complaints have been heard of the dumping of town refuse in country places, and many a village creates a noisome corner of its own for the disposal of tin cans, old boots, and other things not only unsightly but perilous to health. It is hopeless to expect to get rid of the fly menace so long as decaying organic matter is heaped up for long periods. Just as water-carriage is the only reasonable means of removing sewage, so is burning at present the only really practicable and sanitary method of destroying "dust." Some great towns have found it possible to derive energy from the incinerator, but this method of dealing with town refuse is still in its infancy—like so many other matters closely connected with the public health. Were all our sanitary methods on a par with our dealings with the contents of dust-bins we should have to face scores of devastating epidemics every year.

### PREVENTION OF FOG.

SMOKE-LADEN fog is so serious a menace to health that the return to activity, after a period of suspended animation, of the Smoke Abatement League is one more indication of public anxiety to see this troublesome difficulty dealt with more seriously. Fogs resulting from atmospheric conditions, the presence of large bodies of water, and other naturally predisposing causes, are unlikely to be got rid of just at present, though science may yet be equal even to that task. But experience shows that fogs made dense by smoke and heavily-laden with deleterious particles can be mitigated to a far greater extent than at one time seemed possible. London, a peculiarly fog-ridden city, is a fogless paradise compared to its condition a generation ago, partly because of the extensive use of smoke-consuming apparatus, and partly because of the spread of methods of warming which do not involve the immediate consumption of coal. We fancy that the anti-smoke propaganda would make more rapid progress if the propagandists ceased their attacks upon the domestic open fire. That is the ark of the Briton's Covenant; to him a few fogs would be dearly saved if they involved the hugging of the heartless and unresponsive radiator. Moreover, these artificial methods are almost invariably overdone. Houses require not to be heated, but warmed, and there are better means of smoke-prevention than robbing the Briton of his birthright—the cheerful, companionable, romantic open fire. The people of these islands, both men and women, have the best complexions in the world; the Americans, the most over-heated of peoples, have perhaps the worst. By all means let us consume our smoke before it can reach the outer air, but let us not place ourselves upon the level of the unhappy fellow-creatures whose misfortune it is not to dwell in what is, after all, the best climate in the world.

### SALVING THE CLOWN.

IN England pantomime is moribund and its fearful joy, the clown, will apparently soon be extinct. But, even as good Americans, when they die, go to Paris, so, it would seem, does our despised and disregarded English clown emigrate to America when there is no longer any scope in this island for the red-hot poker, and the final sausage has been stolen from the last pork butcher. In the land of the free he undergoes a marvellous translation. He is no longer a thirsty practical joker, always dodging the police and getting innocent people into trouble, but a Federal Official paid and clothed in his characteristic costume by the United States Government. Instead of the traditional poker he is provided with a weighing machine, and as "Cho-Cho, the Health Clown," he weighs all and sundry, and tells them how far they fall short of, or exceed, "what they should weigh in health." Other functions in relation to the public health he, no doubt, performs, but this is obviously his most outstanding duty. His special appeal is to youth, and the United States Public Health Service no doubt appointed "Cho-Cho" because they realise that if people are to be taught the laws of health they cannot be caught too young. We hear nothing of the "patter" with which the weighing is presumably accompanied, though that is obviously as important as the accuracy of the weighing-machine, for was there not once a physician who advised Grimaldi, when he was low-spirited, to go and listen to his own jokes on the stage? Meanwhile it will be a consolation to clowns who see their livelihood departing to know that they may yet take part in a transformation scene in real life.

### WHO SHALL SURVIVE?

"A PHYSICIAN to a London Hospital" raises this vital question in discussing the new treatment for diabetes by means of Insulin. Insulin is the extract of certain cells in the sweetbread or pancreas. The latter organ consists of two parts so intermingled that it is not possible to separate them. The larger part, which may be a hundred times as great as the smaller, has as its function the elaboration of substances essential to digestion. It is the smaller part which makes Insulin, a substance essential to life. At the death of an animal and the removal of the pancreas, the Insulin is destroyed rapidly by the digestive ferments formed by the larger part; therefore the pancreas must be minced and the extractive process begun within a few minutes of death. Hence the laboratory and the abattoir must be close together. So far as is known at present, one pancreas suffices for two doses only. The cost of each dose must be great, and the physician whose views we have thus summarised suggests £250 a year as the annual cost of treating a case of *diabetes mellitus*, and three hundred of these patients die in Great Britain every month! Counsels of perfection suggest that treatment should be given to some 5,000 people annually, and the question arises who should be chosen to be saved through Insulin. Few would care to have the responsibility of selecting cases for treatment from any other than a medical standpoint, but the physician referred to suggests that preference should be given to ex-Service men. No one will quarrel with that; but we must trust to early treatment greatly to reduce the number of cases, and to research to find alternative, or, conceivably, more readily practicable ways of dealing with the disease in its more advanced stages. Meanwhile, it is announced by the Medical Research Council that a number of Hospitals are being supplied with Insulin, and that it is hoped to issue supplies generally in a few weeks' time.

### TUBERCULOSIS STATISTICS.

TOO often statistics in regard to patients who have been undergoing treatment for tuberculosis fail to afford any information of real value. Reports as a rule indicate the number of patients discharged from sanatoria with working capacity partly restored, or who have left prematurely, with some account of the deaths within the year covered by the report, and particulars of dispensary attendances. The difficulties of tracing the subsequent histories of patients are great. It is doubtless for this reason that reviews of results over long periods are so rarely available. Such a review, therefore, as that in the recently published Report of the Medical Officer of Health for Newcastle-upon-Tyne for the year 1921 is a useful contribution to the information on this subject. In this Report, Dr. W. H. Dickinson, the Tuberculosis Medical Officer, brings under review all cases of tuberculosis notified in the ten years 1912-1921. Dr. Dickinson in his main classification of results is careful to avoid any such term as "still living." The classes are "Known to be dead" and "Not known to be dead," and the figures are sufficiently depressing. Of the lung cases, 3,799 were known to be dead, and 2,674 not known to be dead. For the non-pulmonary forms of tuberculosis the figures are 1,187 and 1,533 respectively; 1,055 out of the 1,187 died in the year of notification. It is disturbing to read with regard to deaths occurring in the city from pulmonary tuberculosis (after illness with an average duration of 31 months) that 43·9 per cent. of the patients had either not been notified prior to death (17·2 per cent.), or died within three months of notification (26·7 per cent.), for whom in consequence little or nothing could be done. The figures for non-pulmonary forms of tuberculosis are even worse, for in nearly 50 per cent. the disease had not been notified prior to death. Other particulars with regard to family history, house accommodation, and the distribution of the disease in the various wards of the city are given —particulars which cannot fail to be useful in the event of an inquiry the need for which we urge elsewhere.

### ACCIDENTS AND HURRY.

FOR many years the aim of protective legislation has been direct removal of industrial risk. Until quite recently the impersonal factor has claimed entire attention and, as a direct result, safety devices, machinery guards and the like have reached a commendable degree of perfection. Now, however, a new phase of research has arisen—the study of personal factors. The work is still in its infancy, but when one reads that at least 60 per cent. of all accidents can be traced to a purely personal cause no doubt remains as to its ultimate value. The Industrial Research Fatigue Board is already realising that fatigue as such exerts a relatively minor influence in accident causation. Far more important is the speed at which the work is done. The greater the haste the more frequent are accidents of all kinds. This condemnation of speed is to be interpreted somewhat differently in different spheres of life, but the moral is the same for all. In the language of the factory, if the number of workers and their hours remain the same, you can accelerate output only at the expense of greater accident risk. Rate of movement is the crucial point. Hurry multiplies his risk whether, for example, a man in a busy street is only chasing an omnibus or actually driving it. The excessive activity of modern existence, the rush of the towns, is tending steadily in one direction—man is daily less safe from injury. And not only this. Statistics of accidents in general show that a definite rise occurs at times when the community is in a low state of health, as during an influenza outbreak. Then hurry becomes a more dangerous enemy than ever. The man who habitually hurries may find his life brighter in consequence, but he is very apt to curtail it.

### THE DIPLOMA IN PUBLIC HEALTH.

NEW regulations of the General Medical Council governing the admission of medical men to the Diploma in Public Health would not appear at first sight to be of interest outside medical circles. But a correspondent of the "British Medical Journal" in a recent letter raises a question which has a significance for all who are interested in public health administration. He points out that the Council's new rules, which will apply from January, 1924, onwards, require a curriculum for the D.P.H. extending over not less than twelve months, and six months' practical training under a medical officer of health, and that at least two years must elapse, after obtaining a registrable qualification, before admission to the final examination for the Diploma. These requirements are so much more stringent than those at present in force as to suggest that any medical man who is in future to obtain a qualification must make up his mind while still a medical student that he intends to make his career in public health. No longer will the general practitioner be able to work for his D.P.H. while actually engaged in general practice, as is often done nowadays by those within reach of the necessary facilities. What is to be the result ? Is the part-time medical officer of health in the small towns and rural districts to hold his appointment without any special public health qualification ? Or is he to be abolished and his duties merged in those of full-time assistant medical officers of health ? Apart from these questions, it is essential that general practitioners who have no intention of seeking part-time appointments, should not be discouraged from regarding a course in public health as part of their necessary and normal equipment, as many of them do at present. The suggestion made in the letter to which we refer, that there should be a separate qualification, available to general practitioners under conditions not differing widely from those which at present obtain, as well as that conforming to the new higher standard, merits the close examination of the responsible authorities.

### THE CARE OF THE TEETH.

IT is being realised by all sections of the community with increasing force that dental treatment belongs to the sphere of preventive medicine. The family doctor is too often culpably slow in appreciating the fact, and many of us have had painful experience of prolonged illness where an earlier discovery by the doctor of the septic condition of the mouth might have saved a good deal of suffering and expense. In the belief that every form of service which properly falls within the definition of preventive medicine is essentially one for public organisation, we have no doubt that the Minister of Health will watch with interest the progress of Sir Philip Sassoon's Adult Dental Clinic at Folkestone. The experiment is highly interesting. The main object of the Clinic is the excellent one of carrying on the good work of the school dental clinic when children finish their schooling. The Clinic is, in fact, held on the premises of the School Medical Department, with the consent of the Education Committee, and the school dentist is in charge. The parents of the children are also encouraged to attend. The scale of fees is low, treatment and advice being restricted to persons who are unable to afford the fees of private dentists. We have used the word " experiment " advisedly, in view of the fact that some form of public organisation of dental services is inevitable, whether as part of the National Health Insurance scheme or otherwise. Public organisation, we may note in passing, does not necessarily imply financial assistance from the Exchequer. We hope that other public-spirited men of means will see their way to follow in Sir Philip Sassoon's footsteps. The lessons to be learned from their efforts, as well as from the schemes which some of the Approved Societies have started on a small scale, out of surplus Insurance funds, will be invaluable when an effective organisation of dental services as part of the general Health Services of the community becomes practicable.

### THE ILLEGITIMATE CHILD.

A VERY large number of illegitimate children are born in this country every year, and the death-rate among them is far greater than among children born in wedlock. This is almost inevitable. It is not to the interest of either parent to lavish care upon unwanted children, and in any case the present standard of affiliation payments is too low to enable the mother to nurture them properly. Legitimacy and illegitimacy are alike to Nature, and it is for the State to insist that the base-born enjoy the same opportunities of healthy maturity as those which enter the world with the protection of social sanctions. We are glad, therefore, to know that the Home Secretary has assured a deputation from the National Council for the Unmarried Mother and her Child of his sympathy with its objects, and his agreement with the proposed raising of the contribution. Another important object which the Council have in view is legitimation subsequent to birth. England is believed to be almost the only civilised country in which *legitimatio per subsequens matrimonium* does not prevail. It exists in Scotland without harm to morality; and, in any case, it is a scandal that an illegitimate child's status should suffer all through life notwithstanding the marriage of its parents. Last Session the Government had a Bill for righting this wrong, but it reached the Lords too late, and technical flaws have been found in it. We sincerely hope that the new Administration will be able to find time, without serious delay, to remove this heavy injustice, remembering always that the legitimate have a better chance of vigorous life than the illegitimate.

### THE MEDICAL OFFICER'S YEARLY OPPORTUNITY.

THE Minister of Health has invited the attention of all Local Authorities to the requirement of Article 14 (3) of the Sanitary Officers' Order, 1922, which provides that every Medical Officer of Health is required each year to make a Report to the Local Authority which he serves, on the sanitary circumstances, the sanitary administration and the vital statistics of his district. The report must contain such information as may be required by the Minister and also such other matters as the Medical Officer may consider it desirable to report. The requirements of the Minister, in fact, extend not only to the tabulation of information in the form set out in the Appendix to the circular (this uniformity being essential for purposes of comparison between one area and another), but also to a statement of conditions especially prejudicial to the health of the area, or of any noteworthy occurrences in Public Health administration in the area during the year. This report—apart from the mere tabulation of facts and figures, which usually make rather dry reading—is the Medical Officer's opportunity of reaching the public and stirring up public interest and enthusiasm, especially if the noteworthy occurrences and conditions are presented in a readable narrative form. It is an opportunity of which in their zeal for the Public Health Medical Officers will, we hope, take the fullest advantage.

